# Association between dynapenic obesity and risk of cardiovascular disease: The Hisayama study

**DOI:** 10.1002/jcsm.13564

**Published:** 2024-10-08

**Authors:** Yu Setoyama, Takanori Honda, Takahiro Tajimi, Satoko Sakata, Emi Oishi, Yoshihiko Furuta, Mao Shibata, Jun Hata, Takanari Kitazono, Yasuharu Nakashima, Toshiharu Ninomiya

**Affiliations:** ^1^ Department of Epidemiology and Public Health, Graduate School of Medical Sciences Kyushu University Fukuoka Japan; ^2^ Department of Orthopaedic Surgery, Graduate School of Medical Sciences Kyushu University Fukuoka Japan; ^3^ Center for Cohort Studies, Graduate School of Medical Sciences Kyushu University Fukuoka Japan; ^4^ Department of Medicine and Clinical Science, Graduate School of Medical Sciences Kyushu University Fukuoka Japan; ^5^ Department of Health Care Administration and Management, Graduate School of Medical Sciences Kyushu University Fukuoka Japan

**Keywords:** Body mass index, Cardiovascular disease, Dynapenic obesity, Handgrip strength

## Abstract

**Background:**

Dynapenic obesity is a condition characterized by high adiposity levels combined with muscle dysfunction. Although high adiposity and muscle loss/dysfunction are thought to synergistically increase the risk of cardiovascular disease (CVD), few studies have addressed the association between dynapenic and sarcopenic obesity and CVD. We aimed to investigate the association of dynapenic obesity with incident CVD events using the data from a population‐based prospective longitudinal study in Japan.

**Methods:**

A total of 2490 community‐dwelling Japanese aged 40–79 years (42.5% males, mean age 57.7 ± 10.6 years) without a history of CVD were followed up for a median of 24 years. Handgrip strength was classified as low, medium, or high by age‐ and sex‐specific tertiles. Body mass index (BMI) levels were categorized as lean (<18.5 kg/m^2^), normal (18.5–24.9 kg/m^2^), or obese (≥25.0 kg/m^2^). Dynapenic obesity was defined as having both low handgrip strength and obesity. The outcomes were defined as the first‐ever development of CVD (defined as stroke or coronary heart disease). The hazard ratios (HRs) and their 95% confidence intervals (CIs) for the development of CVD were estimated using a Cox proportional hazards model, in which participants with high handgrip strength and normal BMI were used as a reference group. Mediation analyses used serum high‐sensitivity C‐reactive protein (hs‐CRP) and homeostatic model assessment for insulin resistance (HOMA‐IR) as mediators.

**Results:**

During the follow‐up period, 482 participants developed CVD events (324 cases of stroke and 209 of coronary heart disease). The multivariable‐adjusted risk of CVD increased significantly among participants with dynapenic obesity compared with the reference group (HR 1.49, 95% CI 1.03–2.17). An analysis by age groups showed a further increase in the risk of CVD among participants with dynapenic obesity aged less than 65 years (HR 1.66, 95% CI 1.04–2.65). In mediation analyses for participants aged less than 65 years, serum hs‐CRP was shown to be a significant mediator explaining 13.8% of the association between dynapenic obesity and the development of CVD, while HOMA‐IR explained 12.2% of this relationship.

**Conclusions:**

Dynapenic obesity was a significant risk factor for the development of CVD in a general Japanese population. This association was more pronounced among those aged <65 years. Inflammation, and possibly glucose metabolism, might partly mediate this association. Our findings suggest that preventing muscle dysfunction as well as appropriate weight control, especially in middle‐age, are important for preventing the development of CVD.

## Introduction

Sarcopenia is a multifaceted condition characterized by age‐related progressive loss of skeletal muscle mass, muscle strength, and/or physical performance and is widely recognized to be associated with an elevated risk of various adverse health outcomes, such as falls, fractures, physical dysfunction, and mortality.^1^, ^2^, ^3^ Dynapenia is a similar but distinct condition in which decreased muscle strength occurs regardless of skeletal muscle mass.^4^, ^5^ Recent observational findings have suggested that the decline of muscle strength (i.e., dynapenia) was more closely associated with functional decline and mortality than that of muscle mass, suggesting that its predictive ability may be superior to that of sarcopenia.^4^, ^5^, ^6^, ^7^

Dynapenic (and sarcopenic) obesity, which encompasses both dynapenia (sarcopenia) and excess adiposity, has garnered attention in recent years.^8^, ^9^, ^10^ It is thought that reduced physical activity resulting from obesity contributes to metabolic alteration, while altered skeletal muscle energy metabolism further exacerbates obesity.^11^, ^12^, ^13^ Consequently, adiposity and decreased muscle function in dynapenic and sarcopenic obesity are thought to synergistically increase the risk of functional impairments and mortality.^14^, ^15^, ^16^ Notably, obesity is a well‐established risk factor for cardiovascular disease (CVD),^17^, ^18^ and there have also been recent reports of an association between muscle weakness and the development of CVD.^19^ Therefore, it could be hypothesized that people with dynapenic obesity are at a higher risk of developing CVD. To date, few studies have addressed the prospective association between dynapenic (and sarcopenic) obesity and the risk of CVD, and those studies that have examined this association have inconsistent findings, as well as relatively short follow‐up periods and exclusively Western study populations.^20^, ^21^, ^22^

The present study aims to investigate the association of dynapenic obesity, defined based on handgrip strength and body mass index (BMI), with the development of CVD using the 24‐year follow‐up data from a prospective population‐based cohort study in Japan.

## Methods

### Study population

The Hisayama Study, an ongoing community‐based cohort study on chronic diseases including CVD, is conducted in the town of Hisayama, which is located in the Fukuoka metropolitan area in southwest Japan. Details of the survey were reported previously.^23^, ^24^ In 1988, 2587 residents aged between 40 and 79, constituting approximately 80.2% of the total population within this age bracket, participated in the baseline health examination. After excluding 85 subjects with a history of CVD (as defined by the presence of stroke or coronary heart disease) and 4 subjects who died before starting follow‐up, as well as 7 subjects with missing data on handgrip strength and 1 subject with missing data on BMI, we enrolled the remaining 2490 participants (1059 men and 1431 women) in the present study. Figure S1 shows the flowchart of the enrolment. Because this study had a closed cohort design, there was no additional enrolment after the baseline examination. The present study was approved by the Institutional Review Board for Clinical Research (approval no. 23061‐02) of Kyushu University. The participants provided oral or written informed consent.

### Outcomes

The primary outcomes of the present study were the first‐ever development of CVD and its subtypes, stroke and coronary heart disease (CHD). The details of each definition were described previously.^23^ In brief, stroke was defined as a sudden onset of nonconvulsive and focal neurological deficit persisting for more than 24 h. CHD included acute or silent myocardial infarction, angioplasty or coronary artery bypass surgery, or sudden cardiac death within 1 h of symptom onset.

### Follow‐up survey

The participants were followed up prospectively from December 1988 to November 2012 through repeated health examinations (a median of 24 years, interquartile range of 15–24 years). The details were reported elsewhere.^23^, ^24^ For those who resided in the town, information on suspected cases was constantly collected from the staff of the town office, general practitioners, and healthcare facilities in and around the town in addition to the annual health examinations. For individuals who moved out of the town, regular surveys were carried out by telephone and mail, as well as by enquiries to the relevant healthcare facilities. When we obtained information about a new or suspected CVD event, we examined and evaluated the detailed clinical information to determine whether the event met the definition of an outcome. Additionally, when a participant died, we conducted a comprehensive review of all accessible clinical data. This involved conducting interviews with the attending physician as well as the family members of the deceased and making efforts to obtain permission from the family for an autopsy. During the follow‐up period, a total of 961 participants died, of whom 645 (67.1%) underwent autopsy. No participants were lost to follow‐up.

### Definition of dynapenic obesity

At the baseline examination, a public health nurse measured the handgrip strength of participants using a Smedley hand dynamometer (MIS; Tokyo). We measured the participant's maximum handgrip strength on each side twice, and the maximum of the four measurements was used.^25^ Because it is known that handgrip strength differs between men and women and declines linearly with aging, we controlled for potential sex‐ and age‐related confounders by classifying participants into three groups according to their sex‐specific tertiles of handgrip strength (i.e., low, medium, and high) for each 10‐year age group (Table S1). We measured body height and weight in light clothing without shoes and calculated BMI at baseline. We categorize the participants as lean (<18.5 kg/m^2^), normal (18.5–24.9 kg/m^2^), or obese (≥25.0 kg/m^2^) based on their BMI levels.^26^ We also divided the participants into nine groups based on their combination of handgrip‐strength (three groups) and BMI (three groups). Participants with both obesity and low handgrip strength were defined as having dynapenic obesity.

### Other risk factors

At the baseline examination, participants completed questionnaires about their medical history, treatment for hypertension and diabetes, alcohol intake, smoking habits, and regular exercise habits. We classified alcohol intake and smoking habits as current use or not. Participants were also asked about their leisure‐time regular exercise, and the results were dichotomized as exercising at least three times a week (regular exercise habit) or not. We also estimated the amount of physical activity during the leisure‐time exercise activities by multiplying the intensity (metabolic equivalents [METs]) and duration (hours per week) of each activity.^27^ Resting blood pressure, taken as the mean value of three measurements in the sitting position, was also analysed, and hypertension was defined as a blood pressure level of ≥140/90 mmHg and/or current treatment with antihypertensive agents.^28^ ECG abnormalities included left ventricular hypertrophy (Minnesota Code, 3–1), ST depression (4–1, 2, 3), or atrial fibrillation or atrial flutter (8–3). Plasma glucose levels were determined by the glucose‐oxidase method, and diabetes mellitus was defined as either a fasting plasma glucose level ≥126 mg/dL, postprandial, a 2‐h post‐load glucose level ≥200 mg/dL or current treatment with oral hypoglycemic agents or insulin.^29^ Serum total and high‐density lipoprotein (HDL) cholesterol levels were determined enzymatically. Serum high‐sensitivity C‐reactive protein (hs‐CRP) levels were measured on a Behring Nephelometer BN‐100 (Behring Diagnostics) using a modified version of the Behring Latex‐Enhanced CRP assay. The homeostatic model assessment for insulin resistance (HOMA‐IR) was calculated as fasting glucose (mg/dL) × fasting insulin (μU/ml) /405.^30^

### Statistical analysis

To examine the impact of dynapenic obesity on the incidence of CVD and its subtypes, all analyses were conducted in participants with high handgrip strength and normal BMI as a reference group. Descriptive statistics were computed, and the group differences were tested using linear and logistic regression against the reference group.^31^

We calculated the age‐ and sex‐adjusted incidence rates (per 1000 person‐years) of CVD by the direct method based on the total population examined stratified by 10‐year age group and sex. The hazard ratios (HRs) and 95% confidence intervals (CIs) for the risk of incident CVD were estimated using a Cox proportional hazards model. In multivariable‐adjusted analyses, we adjusted for potential confounders including age, sex, hypertension, diabetes, serum total cholesterol level, serum HDL cholesterol level, ECG abnormalities (presence of either left ventricular hypertrophy, ST depression, atrial fibrillation, or atrial flutter), current alcohol intake, smoking habits, and regular exercise (exercising three or more times per week). We also conducted subgroup analyses by age (<65 years and ≥65 years) and by sex for the association between dynapenic obesity and CVD risk: heterogeneity in the association by age and sex was tested by adding a cross‐product term with the relevant Cox model. We used an age of 65 years to distinguish middle‐aged and older subgroups, as this is the widely accepted cut‐off for defining older individuals both in Japan and internationally.^32^, ^33^ Given that each of decreased handgrip strength and obesity is individually recognized as a risk factor for developing CVD, we conducted additional analyses using only handgrip strength or only BMI as the exposure variable in order to assess the independent effects of each component. For the sensitivity analyses, we considered competing risks of death using the Fine–Gray subdistribution hazards model.^34^ In addition, to determine whether the results differed when alternative measures were used for muscle and obesity, we conducted the analysis using waist circumferences instead of BMI as a measure of abdominal obesity, where the cut‐off for abdominal obesity was defined as a waist circumference of ≥90 cm for men and ≥80 cm for women.^26^ We then performed an additional sensitivity analysis using the Asian Working Group for Sarcopenia 2019 (AWGS 2019) cut‐offs of <28 kg for men and <18 kg for women in order to dichotomize handgrip strength levels.^2^

To examine whether chronic inflammation and increased insulin resistance mediate the association between dynapenic obesity and the risk of CVD as suggested in previous reports,^11^, ^12^, ^13^ we performed causal mediation analyses using the % mediation macro referencing Valeri & Vanderweele.^35^ We used serum hs‐CRP levels as a marker of chronic inflammation and the HOMA‐IR as a marker of insulin resistance. Because the distributions of serum hs‐CRP and HOMA‐IR were skewed, we performed a logarithmic transformation before the analysis.

The SAS software package version 9.4 (SAS Institute, Cary, NC) was used for all analyses. For statistical tests, a two‐sided *P* value of <0.05 was considered significant.

## Results

The number and proportion of the participants classified by handgrip strength levels and BMI levels are shown in Table 1. Of 2490 participants, 603 (24.2%) were classified as being obese, and 135 (5.4%) were classified as having dynapenic obesity at the baseline examination. Table 2 shows the baseline characteristics according to the nine groups by handgrip strength levels and BMI levels. The mean age of all participants was 57.7 years, and 42.5% were male. Compared with the reference group (participants with high handgrip strength and normal BMI), the participants with dynapenic obesity had higher mean values of serum total cholesterol level, higher median values of serum hs‐CRP level and HOMA‐IR, and higher proportions of hypertension and diabetes mellitus. On the other hand, they had a lower mean value of serum HDL cholesterol and physical activity (METs‐h/week) and a lower proportion of participants with regular exercise habits. The baseline characteristics of each handgrip and BMI category are shown in Table S2.

**Table 1:** Number and proportion of participants by handgrip strength and BMI levels

Handgrip strength levels	BMI levels		
	Lean (BMI < 18.5 kg/m^2^)	Normal weight (BMI 18.5–24.9 kg/m^2^)	Obese (BMI ≥ 25.0 kg/m^2^)
Low	84 (3.4)	541 (21.7)	135 (5.4) (dynapenic obesity)^a^
Medium	47 (1.9)	588 (23.6)	191 (7.7)
High	32 (1.3)	595 (23.9) (reference)^b^	277 (11.1)

All values are shown as the number of participants at risk (%). Handgrip strength levels were classified according to age‐ and sex‐specific tertiles.

BMI, body mass index.

^a^ Dynapenic obesity was defined as having both low handgrip strength and obesity (BMI ≥ 25.0 kg/m^2^).

^b^ Reference group included the participants with both high handgrip strength and normal weight (BMI 18.5–24.9 kg/m^2^).

**Table 2:** Baseline characteristics of participants according to handgrip strength and BMI levels in 1988

BMI levels	All participants	Lean (BMI < 18.5 kg/m^2^)			Normal weight (BMI 18.5–24.9 kg/m^2^)			Obese (BMI ≥ 25.0 kg/m^2^)		
Handgrip strength levels		High	Medium	Low	High (reference)	Medium	Low	High	Medium	Low (dynapenic obesity)
	(*n* = 2490)	(*n* = 32)	(*n* = 47)	(*n* = 84)	(*n* = 595)	(*n* = 588)	(*n* = 541)	(*n* = 277)	(*n* = 191)	(*n* = 135)
Handgrip strength, median (IQR), kg	29.3 (23.0–39.0)	30.0 (25.5–36.5)**	25.0 (19.5–36.5)**	21.8 (15.0–30.0)**	33.0 (28.0–45.0)	27.5 (23.5–39.3)**	22.5 (18.0–33.0)**	35.0 (29.5–50.0)**	28.5 (23.0–40.0)**	22.0 (17.0–35.0)**
BMI, median (IQR), kg/m^2^	22.8 (20.7–24.9)	17.4 (16.8–18.1)**	17.7 (17.0–18.1)**	17.8 (16.9–18.2)**	22.4 (21.1–23.8)	22.1 (20.6–23.5)*	21.7 (20.2–23.0)**	26.6 (25.7–28.1)**	26.5 (25.8–27.5)**	26.4 (25.6–28.1)**
Age, mean (SD), years	57.7 (10.6)	63.1 (10.8)**	65.0 (10.3)**	61.7 (11.4)**	57.1 (10.8)	57.6 (10.5)	58.4 (10.6)*	55.5 (9.6)*	57.1 (10.1)	57.7 (9.8)
Men, %	42.5	28.1	42.6	45.2	41.5	43.4	43.4	44.0	43.5	37.0
Hypertension, %	39.3	25	34	34.5	33.9	37.6	33.5	56.3**	51.8**	49.6**
Diabetes mellitus, %	11.9	12.5	8.5	10.7	6.7	11.1**	12.2**	17.0**	18.3**	20.0**
Serum total cholesterol, mean (SD), mg/dL^a^	206.8 (40.0)	213.7 (41.5)	194.4 (31.7)*	196.4 (38.1)*	207.6 (40.2)	205.0 (42.2)	203.3 (44.6)	212.7 (41.5)	210.4 (41.4)	217.7 (42.3)*
Serum HDL cholesterol, mean (SD), mg/dL^a^	50.5 (11.7)	58.5 (14.1)**	53.4 (10.9)	55.3 (14.3)**	51.6 (11.7)	50.7 (11.2)	51.5 (12.1)	46.9 (10.4)**	46.9 (10.7)**	46.9 (10.6)**
Electrocardiogram abnormalities, %	16.3	6.3	27.7*	20.2	15.6	14.5	20.7*	13.4	14.7	13.3
Current alcohol intake, %	31.5	21.9	23.4	33.3	31.4	31.1	33.1	34.3	29.8	27.4
Current smoking habits, %	25.4	31.3	42.6**	31.0	23.0	26.0	28.5*	20.9	23.0	23.0
Regular exercise of ≥3 times/week, %	9.9	9.4	10.6	9.5	11.1	11.4	10.0	8.7	6.8	5.2*
Physical activity, median (IQR), METs‐h/week^b^	0.00 (0.00–0.00)	0.00 (0.00–4.40)	0.00 (0.00–0.00)	0.00 (0.00–0.00)	0.00 (0.00–2.15)	0.00 (0.00–0.80)**	0.00 (0.00–0.00)*	0.00 (0.00–1.60)	0.00 (0.00–0.00)	0.00 (0.00–0.00)*
Serum hs‐CRP, median (IQR), mg/L^c^	0.44 (0.21–1.00)	0.21 (0.12–0.45)	0.20 (0.12–0.62)	0.25 (0.13–0.69)	0.37 (0.18–0.78)	0.40 (0.20–1.03)*	0.42 (0.20–0.94)*	0.60 (0.30–1.24)**	0.64 (0.34–1.22)**	0.79 (0.34–1.49)**
HOMA‐IR, median (IQR)^d^	1.4 (1.0–2.1)	1.0 (0.8–1.5)*	1.0 (0.7–1.3)**	0.9 (0.7–1.2)**	1.3 (0.9–1.8)	1.3 (0.9–1.8)	1.3 (0.9–1.8)	2.2 (1.4–3.0)**	2.1 (1.6–3.0)**	2.2 (1.4–2.8)**

BMI, body mass index; HDL, high‐density lipoprotein; HOMA‐IR, homeostatic model assessment for insulin resistance; hs‐CRP, high‐sensitivity C‐reactive protein; IQR, interquartile range; METs, metabolic equivalents; SD, standard deviation.

Handgrip strength levels were classified according to age‐ and sex‐specific tertiles. BMI levels were classified as lean (<18.5 kg/m^2^), normal weight (18.5–24.9 kg/m^2^), and obese (≥25.0 kg/m^2^).

^a^ Missing in 1 participant.

^b^ Missing in 35 participants.

^c^ Missing in 21 participants.

^d^ Missing in 65 participants.

* *P* < 0.05.

^**^ *P* < 0.01 versus a reference group (participants with a high handgrip strength and BMI of 18.5–24.9 kg/m^2^).

### Main analysis

**Figure 1:**
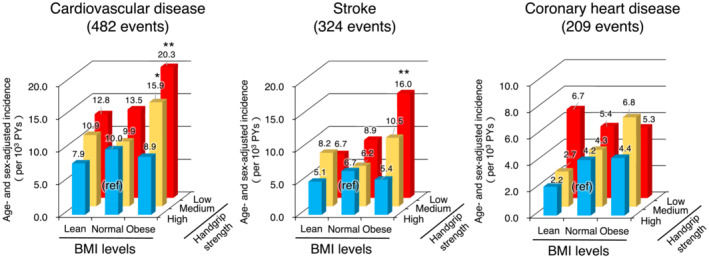
Age‐ and sex‐adjusted incidence of cardiovascular disease and its subtypes according to BMI and handgrip strength classification, 1988–2012. *P < 0.05, **P < 0.01 versus a reference group (participants with a high handgrip strength and BMI of 18.5–24.9 kg/m^2^). BMI, body mass index; PYs, person‐years.

Figure 1 shows the age‐ and sex‐adjusted incidence rates of CVD, stroke, and CHD. Additional details, including the 95% CIs of incidence, are shown in Table S3. During the follow‐up period, 482 participants developed a first‐ever CVD event, including 324 cases of stroke and 209 cases of CHD. The incidence rate of CVD (per 1000 person‐years) was 20.3 (95% CI 12.8–27.7) in participants with dynapenic obesity and 10.0 (95% CI 8.1–12.0) in the reference group, showing a significant group difference (*P* = 0.001). Similarly, the incidence rate of stroke was significantly higher in participants with dynapenic obesity than in participants of the reference group (16.0 vs. 6.7 per 1000 person‐years, *P* = 0.002). In contrast, there was no significant group difference in the incidence of CHD across the nine categories. As shown in Table 3, the multivariable‐adjusted HRs for the development of CVD increased significantly among participants with dynapenic obesity (HR 1.49, 95% CI 1.03–2.17), compared with the reference group. Similar associations were observed for stroke (HR 1.65, 95% CI 1.06–2.57) but not for CHD (HR 1.19, 95% CI 0.65–2.20).

**Table 3:** Multivariable‐adjusted hazard ratios of the development of cardiovascular disease and its subtypes according to handgrip strength and BMI classification (*n* = 2490), 1988–2012

BMI levels	Lean (BMI < 18.5 kg/m^2^)			Normal weight (BMI 18.5–24.9 kg/m^2^)			Obese (BMI ≥ 25.0 kg/m^2^)		
Handgrip strength levels	High	Medium	Low	High	Medium	Low	High	Medium	Low (dynapenic obesity)
Cardiovascular disease									
No. of events/at risk	5/32	13/47	18/84	105/595	100/588	108/541	45/277	49/191	39/135
HR (95% CI)	0.81 (0.32–2.00)	1.37 (0.76–2.45)	1.19 (0.72–1.97)	1.00 (reference)	0.94 (0.71–1.23)	1.17 (0.89–1.53)	0.83 (0.58–1.18)	1.34 (0.95–1.88)	1.49 (1.03–2.17)*
Stroke									
No. of events/at risk	3/32	10/47	10/84	71/595	65/588	75/541	28/277	33/191	29/135
HR (95% CI)	0.71 (0.22–2.28)	1.47 (0.75–2.88)	0.93 (0.48–1.82)	1.00 (reference)	0.91 (0.65–1.27)	1.21 (0.87–1.67)	0.78 (0.50–1.21)	1.33 (0.87–2.01)	1.65 (1.06–2.57)*
Coronary heart disease									
No. of events/at risk	2/32	3/47	10/84	46/595	45/588	45/541	23/277	21/191	14/135
HR (95% CI)	0.74 (0.18–3.10)	0.77 (0.24–2.50)	1.56 (0.78–3.11)	1.00 (reference)	0.97 (0.64–1.47)	1.09 (0.72–1.64)	0.95 (0.57–1.58)	1.25 (0.74–2.12)	1.19 (0.65–2.20)

The models were adjusted for age, sex, hypertension, diabetes, serum total cholesterol, serum high‐density lipoprotein cholesterol, electrocardiogram abnormalities, current alcohol intake, current smoking habits, and regular exercise. Handgrip strength levels were classified according to age‐ and sex‐specific tertiles. BMI levels were classified as lean (<18.5 kg/m^2^), normal weight (18.5–24.9 kg/m^2^), and obese (≥25.0 kg/m^2^).

BMI, body mass index; CI, confidence intervals; HR, hazard ratio.

* *P* < 0.05 versus a reference group (participants with a high handgrip strength and BMI of 18.5–24.9 kg/m^2^).

Table 4 shows the analyses conducted by the different age groups and sex. The results in participants aged <65 years were similar to those for all participants, with the risk of CVD increasing significantly among participants with dynapenic obesity (HR 1.66, 95% CI 1.04–2.65). In participants aged ≥65 years, the risk of CVD in participants with dynapenic obesity was not elevated compared with the reference group. In the analysis by sex, the CVD risk for participants with dynapenic obesity was HR 1.63 (95% CI 0.95–2.81) for men and HR 1.31 (95% CI 0.77–2.21) for women. Heterogeneity in the association by age groups was suggestive (*P* for heterogeneity = 0.13), but that for the association by sex was not (*P* for heterogeneity = 0.79): subsequent analyses were presented by age. We confirmed that the observed associations were unchanged when the amount of physical activity was included as a covariate instead of the dichotomized variable of regular exercise habits. We therefore used regular exercise habits as an adjusting variable in all analyses, as there were fewer missing values for this parameter.

**Table 4:** Multivariable‐adjusted hazard ratios of development of cardiovascular disease according to handgrip strength and BMI in the subgroups of age or sex, 1988–2012

BMI levels	Lean (BMI < 18.5 kg/m^2^)			Normal weight (BMI 18.5–24.9 kg/m^2^)			Obese (BMI ≥ 25.0 kg/m^2^)		
Handgrip strength levels	High	Medium	Low	High	Medium	Low	High	Medium	Low (dynapenic obesity)
Participants aged <65 years (*n* = 1794)									
No. of events/at risk	2/18	4/20	6/46	60/437	59/440	53/366	32/226	29/142	27/99
HR (95% CI)	0.77 (0.18–3.18)	1.61 (0.58–4.46)	0.96 (0.41–2.24)	1.00 (reference)	0.86 (0.60–1.24)	1.03 (0.71–1.50)	0.85 (0.55–1.32)	1.27 (0.81–1.99)	1.66 (1.04–2.65)*
Participants aged ≥65 years (*n* = 696)									
No. of events/at risk	3/14	9/27	12/38	45/158	41/148	55/175	13/51	20/49	12/36
HR (95% CI)	0.87 (0.26–2.89)	1.42 (0.69–2.96)	1.35 (0.70–2.57)	1.00 (reference)	1.00 (0.65–1.53)	1.39 (0.93–2.07)	0.74 (0.39–1.39)	1.38 (0.81–2.36)	1.18 (0.61–2.27)
Men (*n* = 1059)									
No. of events/at risk	1/9	5/20	12/38	55/247	58/255	57/235	23/122	22/83	19/50
HR (95% CI)	0.26 (0.04–1.95)	1.00 (0.40–2.54)	1.38 (0.73–2.59)	1.00 (reference)	1.03 (0.71–1.49)	1.16 (0.80–1.69)	0.82 (0.50–1.35)	1.22 (0.73–2.02)	1.63 (0.95–2.81)
Women (*n* = 1431)									
No. of events/at risk	4/23	8/27	6/46	50/348	42/333	51/306	22/155	27 108	20/85
HR (95% CI)	1.35 (0.48–3.77)	1.66 (0.77–3.58)	0.89 (0.38–2.10)	1.00 (reference)	0.83 (0.55–1.25)	1.16 (0.79–1.73)	0.81 (0.49–1.35)	1.44 (0.90–2.32)	1.31 (0.77–2.21)

The models were adjusted for age, sex, hypertension, diabetes, serum total cholesterol, serum high‐density lipoprotein cholesterol, electrocardiogram abnormalities, current alcohol intake, smoking habits, and regular exercise. Handgrip strength levels were classified according to age‐ and sex‐specific tertiles. BMI levels were classified as lean (<18.5 kg/m^2^), normal weight (18.5–24.9 kg/m^2^), and obese (≥25.0 kg/m^2^). *P* for heterogeneity was 0.13 for age groups and 0.79 for sex.

BMI, body mass index; CI, confidence intervals; HR, hazard ratio.

* *P* < 0.05 versus a reference group (participants with a high handgrip strength and BMI of 18.5–24.9 kg/m^2^).

### Sensitivity analysis

In the analysis of the components of dynapenic obesity, the participants with low handgrip strength showed a significant increase in the risk of CVD compared with the participants with high handgrip strength among all participants and participants ≥65 years, but this association was not significant among participants aged <65 years (Table S4). The analysis by BMI level showed no significant intergroup differences in those <65 years, those ≥65 years, or all participants (Table S5).

In the sensitivity analyses, significant associations were observed between dynapenic obesity and the risk of CVD while accounting for the competing risk of death (Table S6). Similar results were also observed in the analysis in which obesity was defined by the presence of abdominal obesity (Table S7), as well as in the analysis in which the handgrip strength level was classified by the AWGS2019 cut‐off (Table S8).

### Causal mediation analyses

**Figure 2:**
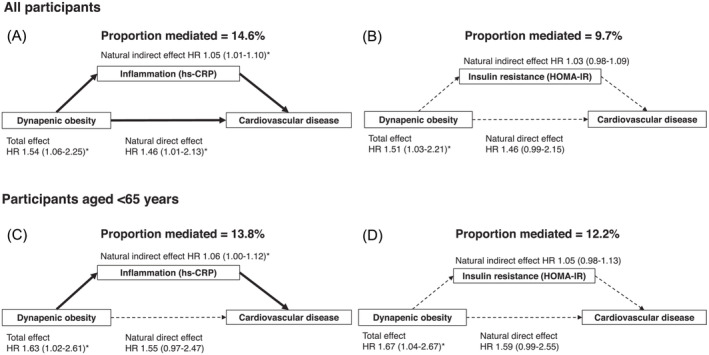
The causal mediation analyses for examining the mediating role of inflammation, as measured by serum hs‐CRP levels, and insulin resistance, as measured by HOMA‐IR, on the association between dynapenic obesity and the development of cardiovascular disease. The analyses were conducted for all participants (A, B) and for those aged <65 years (C, D). *P < 0.05 versus a reference group (participants with a high handgrip strength and BMI of 18.5–24.9 kg/m^2^). HR, hazard ratio; hs‐CRP, high‐sensitivity C‐reactive protein; HOMA‐IR, homeostatic model assessment for insulin resistance.

Results of the causal mediation analyses, in which inflammation or insulin resistance was considered a mediator, for examining the association between dynapenic obesity and the development of CVD are shown in Figure 2. Serum hs‐CRP levels, a measure of inflammation, significantly mediated the association, explaining 14.6% of the association in the total population and 13.8% in participants aged <65 years, respectively. HOMA‐IR as a measure of insulin resistance mediated a similar proportion, explaining 9.7% of the association in the total and 12.2% in the middle‐aged population, although the HR for the indirect effect did not reach statistical significance.

## Discussion

In this prospective study of Japanese community residents, participants with dynapenic obesity, defined by handgrip strength level and BMI level, showed an increased risk of CVD and stroke, even after adjusting for potential confounders. The association was more pronounced in the participants aged <65 years. Our findings suggest that dynapenic obesity is an independent risk factor for the development of CVD, and this may be especially true in the middle‐aged.

As far as we know, only three longitudinal studies have investigated the association between sarcopenic or dynapenic obesity and CVD.^20^, ^21^, ^22^ Our findings were consistent with a prospective study of 452 931 UK residents aged 40–69 years (mean follow‐up of 5.1 years) using the data from the UK Biobank, which showed that participants with sarcopenic (dynapenic) obesity, defined by handgrip strength and BMI level, had a higher risk of development of CVD compared with participants with either decreased handgrip strength or obesity alone.^20^ In contrast to the results of this previous study and our present analysis, a prospective study of 4111 British men aged 60–79 years with a mean follow‐up of 11.3 years found no significant association between sarcopenic obesity, defined by mid‐upper arm circumference and waist circumference, and the risk of CVD.^21^ Another prospective study of 3366 community residents aged 65 years or older in the United States found that sarcopenic obesity when defined by skeletal muscle mass and waist circumference was not associated with risk of CVD but exhibited a marginally significant association with CVD risk when defined by handgrip strength and waist circumference.^22^ Our study included a wide age range of participants, including middle‐aged and older adults, and provided new evidence of the heterogenous consequences of dynapenic and sarcopenic obesity.

The different age ranges across the studies may account for the inconsistencies among the existing evidence. The present study was the first to find an association between dynapenic obesity and the development of CVD in individuals aged <65 years, and we observed a marked association in this age group. Age‐related decline in muscle strength has been reported to begin at about the age of 40, and accumulating evidence has suggested that muscle strength declines more rapidly than muscle mass.^36^ Our findings underscore the need for increased focus on dynapenic obesity in the middle‐aged population. On the other hand, in participants aged over 65 years, we observed that low handgrip strength was significantly associated with the development of CVD, but an excess BMI was not. Previous studies on older adults have provided little support for an association between dynapenic or sarcopenic obesity and the risk of CVD, and many found that the association with mortality was stronger for dynapenia (or sarcopenia) without obesity than for those with obesity.^14^, ^15^, ^16^ Our findings and these results suggest that older adults need increased focus on muscle weakness or dysfunction rather than obesity.

An additional factor contributing to the inconsistent results across the present analysis and these previous studies could be the difference in the diagnostic criteria employed to identify dynapenic or sarcopenic obesity across each study. The use of different criteria was due to the lack of an established definition of sarcopenic obesity.^8^, ^9^, ^10^ Indeed, a previous systematic review reported that the diagnostic criteria of sarcopenic obesity vary widely, with the prevalence of sarcopenic obesity varying up to approximately 40% depending on the population and definition.^37^ In recent years, several associations and consortia have attempted to standardize the definitions and criteria used in the diagnosis of sarcopenic obesity.^38^ The findings from previous prospective studies, along with our present results regarding the association between low handgrip strength and an elevated risk of CVD, suggest that a decrease in muscle strength could play a pivotal role in the development of CVD. The relationship between skeletal muscle mass and clinical outcomes can be particularly unclear in obese individuals, as there is a tendency for relative increases in skeletal muscle mass in these individuals.^39^ Therefore, preventing disproportionate muscle strength decline, as reflected in dynapenic obesity, might be crucial for the prevention of clinical outcomes such as CVD.

The underlying mechanisms linking dynapenic obesity and the development of CVD remain unclear. One potential explanation is the presence of low‐grade systemic inflammation, a well‐established risk factor for CVD, which may be further exacerbated by muscle dysfunction in dynapenic obesity.^10^, ^11^, ^12^, ^13^ The causal mediation analyses in the present study support this, with serum hs‐CRP as a marker of low‐grade systemic inflammation shown as a significant mediator explaining 14.6% of the association between dynapenic obesity and the development of CVD. As such, it is plausible that dynapenic obesity may lead to a more pronounced state of inflammation, thereby contributing to the development of CVD. Also, an increase in insulin resistance and oxidative stress have been considered possible contributing factors to the observed association. As for insulin resistance, the causal mediation analyses showed that HOMA‐IR was a factor explaining 9.7% of the association between dynapenic obesity and the development of CVD. Therefore, insulin resistance could also contribute to the observed association, although the contribution appears weak given that the natural indirect effect was not significant. On the other hand, we could not examine the influence of oxidative stress because data on oxidative stress were not collected at baseline and further studies are needed.

The strengths of the present study include the long‐term longitudinal study design, the use of a community‐based population in Asia, and the wider age range compared with previous studies. In addition, the high participation rates in the screening examination at baseline, complete follow‐up of participants, precise diagnosis of CVD and its subtypes using autopsy findings and available clinical information, and careful evaluation via a series of sensitivity analyses also reinforce the validity of our study. Several limitations also need to be pointed out. First, we defined dynapenia using handgrip strength alone. Future studies should include an assessment of gait speed and/or muscle mass to improve comparability with the relevant studies assessing dynapenic or sarcopenic obesity. Second, the handgrip strength and BMI were only assessed at baseline, so the effect of changes in these exposures is unclear. Third, due to the small sample size, we could not perform a more detailed subgroup analysis, such as for each subtype of CVD. Consequently, future studies with larger sample sizes will be needed to verify our findings. Fourth, the information on physical activity was obtained using an original questionnaire. Therefore, it may be less accurate than validated questionnaires and objective measurements and may not be sufficiently adjusted for physical activity levels. Fifth, we could not obtain the data regarding the presence of resistance exercise in this study. Given that resistance exercise is known to improve skeletal muscle function, future research would benefit from collecting such information. Sixth, we could not eliminate the possibility of selection bias due to non‐participation in the health examination. Finally, this study was conducted in one Japanese community, restricting the generalizability of the conclusions.

## Conclusions

In conclusion, dynapenic obesity, defined by handgrip strength and BMI, was a significant risk factor for the development of cardiovascular disease in a Japanese community population. This association was more pronounced among those aged <65 years. Inflammation, and possibly glucose metabolism, was suggested to partly mediate this association. Our findings suggest that preventing muscle dysfunction as well as appropriate weight control during middle age are important for preventing the development of cardiovascular disease.

## Funding

This study was supported in part by the Ministry of Education, Culture, Sports, Science and Technology of Japan (JSPS KAKENHI grant numbers JP22K07421, JP22K17396, JP23K09692, JP23K09717, JP23K16330, JP23K06787, and JP23K09060); by Health and Labour Sciences Research Grants of the Ministry of Health, Labour and Welfare of Japan (grant numbers JPMH23FA1006, JPMH23FA1022, and JPMH24GB1002); by the Japan Agency for Medical Research and Development (grant numbers JP24dk0207053, JP24km0405209, and JP24tm0524003); by the Japan Science and Technology Agency (grant number JPMJPF2210); by Eli Lilly Japan K.K. (Kobe, Japan); and by the Japanese Orthopaedic Association (JOA‐Subsidized Science Project Research 2022‐2).

## Conflict of interest

All authors declare that they have no conflicts of interest.

## Supporting information


**Table S1.** Ranges of handgrip strength by age‐ and sex‐specific tertiles in 1988.
**Table S2.** Baseline characteristics by handgrip strength level and BMI level in 1988.
**Table S3.** Age‐ and sex‐adjusted incidence (per 1,000 person‐years) of cardiovascular disease and its subtypes according to BMI and handgrip strength classification (n = 2,490), 1988–2012.
**Table S4.** Multivariable‐adjusted hazard ratios of development of cardiovascular disease according to handgrip strength levels, 1988–2012.
**Table S5.** Multivariable‐adjusted hazard ratios of development of cardiovascular disease according to BMI levels, 1988–2012.
**Table S6.** Subdistribution hazard regression (Fine and Grey) models for development of cardiovascular disease accounting for competing risk of death according to handgrip strength and BMI levels, 1988–2012.
**Table S7.** Multivariable‐adjusted hazard ratios of development of cardiovascular disease according to handgrip strength levels and the presence or absence of abdominal obesity, 1988–2012.
**Table S8.** Multivariable‐adjusted hazard ratios of development of cardiovascular disease according to handgrip strength levels classified by the AWGS cut‐off values and BMI levels, 1988–2012.

**Figure S1.** Flowchart of numbers of included and excluded participants.
