# Supplementary material for: Association between dynapenic obesity and risk of cardiovascular disease: The Hisayama study
Source: J Cachexia Sarcopenia Muscle. 2024 Oct 8;15(6):2338–48. doi: 10.1002/jcsm.13564 (PMC11634510; doi:10.1002/jcsm.13564)
Supplement: Supplementary file 1 — Table S1. Ranges of handgrip strength by age‐ and sex‐specific tertiles in 1988. Table S2. Baseline characteristics by handgrip strength level and BMI level in 1988. Table S3. Age‐ and sex‐adjusted incidence (per 1,000 person‐years) of cardiovascular disease and its subtypes according to BMI and handgrip strength classification (n = 2,490), 1988–2012. Table S4. Multivariable‐adjusted hazard ratios of development of cardiovascular disease according to handgrip strength levels, 1988–2012. Table S5. Multivariable‐adjusted hazard ratios of development of cardiovascular disease according to BMI levels, 1988–2012. Table S6. Subdistribution hazard regression (Fine and Grey) models for development of cardiovascular disease accounting for competing risk of death according to handgrip strength and BMI levels, 1988–2012. Table S7. Multivariable‐adjusted hazard ratios of development of cardiovascular disease according to handgrip strength levels and the presence or absence of abdominal obesity, 1988–2012. Table S8. Multivariable‐adjusted hazard ratios of development of cardiovascular disease according to handgrip strength levels classified by the AWGS cut‐off values and BMI levels, 1988–2012. [file JCSM-15-2338-s001.docx]

| **Supplemental Table 1. Ranges of handgrip strength by age- and sex-specific tertiles in 1988** | | | | |
| --- | --- | --- | --- | --- |
| Age, years | Average handgrip strength, kg | Tertiles of handgrip strength, kg | | |
|  |  | Low | Medium | High |
| ***Men*** |  |  |  |  |
| 40–49 | 46.8 | 25.0–43.5  (n = 96) | 44.0–49.5  (n = 101) | 50.0–64.0  (n = 105) |
| 50–59 | 42.2 | 18.0–38.5  (n = 90) | 39.0–44.5  (n = 108) | 45.0–62.0  (n = 119) |
| 60–69 | 38.4 | 12.0–35.5  (n = 86) | 36.0–40.0  (n = 101) | 40.5–62.0  (n = 94) |
| 70–79 | 32.2 | 15.0–29.5  (n = 51) | 30.0–34.5  (n = 48) | 35.0–48.0  (n = 60) |
| ***Women*** |  |  |  |  |
| 40–49 | 28.0 | 3.5–25.5  (n = 106) | 26.0–29.5  (n = 118) | 30.0–42.0  (n = 156) |
| 50–59 | 24.8 | 7.0–22.5  (n = 135) | 23.0–26.5  (n = 133) | 27.0–42.0  (n = 156) |
| 60–69 | 22.4 | 0.5–20.0  (n = 125) | 20.5–24.5  (n = 126) | 25.0–39.0  (n = 132) |
| 70–79 | 17.8 | 3.0–15.5  (n = 71) | 16.0–20.0  (n = 91) | 20.5–30.0  (n = 82) |

| **Supplemental Table 2. Baseline characteristics by handgrip strength level and BMI level in 1988** | | | | | | | | | |
| --- | --- | --- | --- | --- | --- | --- | --- | --- | --- |
|  | Handgrip strength levels | | | p value |  | BMI levels | | | p value |
|  | Low | Medium | High |  |  | Lean | Normal weight | Obese |  |
|  | (n = 760) | (n = 826) | (n = 904) |  |  | (n = 194) | (n = 1,819) | (n = 612) |  |
| Age, mean (SD), years | 58.6 (10.6) | 57.9 (10.5) | 56.8 (10.6) | <0.001 |  | 62.9 (11.0) | 57.7 (10.6) | 56.5 (9.8) | <0.001 |
| Men, % | 42.5 | 43.3 | 41.8 | 0.76 |  | 41.1 | 42.7 | 42.3 | 0.95 |
| Hypertension, % | 36.4 | 40.7 | 40.5 | 0.10 |  | 32.5 | 35.0 | 53.4 | <0.001 |
| Diabetes mellitus, % | 13.4 | 12.6 | 10.1 | 0.03 |  | 10.4 | 9.9 | 18.1 | <0.001 |
| Serum total cholesterol, mean (SD), mg/dL ^a)^ | 205.1 (43.9) | 205.6 (41.6) | 209.4 (40.7) | 0.03 |  | 199.2 (37.6) | 205.3 (42.3) | 213.1 (41.7) | <0.001 |
| Serum HDL cholesterol, mean (SD), mg/dL ^a)^ | 51.1 (12.3) | 50.0 (11.2) | 50.4 (11.7) | 0.29 |  | 55.4 (13.4) | 51.3 (11.7) | 46.9 (10.5) | <0.001 |
| Electrocardiogram abnormalities, % | 19.3 | 15.3 | 14.6 | 0.01 |  | 19.6 | 16.8 | 13.8 | 0.03 |
| Current alcohol intake, % | 32.1 | 30.4 | 32.0 | 0.99 |  | 28.2 | 31.8 | 31.3 | 0.71 |
| Current smoking habits, % | 27.8 | 26.3 | 22.7 | 0.02 |  | 34.4 | 25.8 | 22.1 | 0.002 |
| Regular exercise of ≥3 times/week, % | 9.1 | 10.3 | 10.3 | 0.42 |  | 9.8 | 10.8 | 7.3 | 0.05 |
| Physical activity, median (IQR),  METs-h/week ^b)^ | 0.00  (0.00-0.00) | 0.00  (0.00-0.00) | 0.00  (0.00-1.90) | 0.04 |  | 0.00  (0.00 - 0.00) | 0.00  (0.00 - 0.00) | 0.00  (0.00 - 0.00) | 0.06 |
| Serum hs-CRP, median (IQR), mg/L ^c)^ | 0.43  (0.20–1.04) | 0.46  (0.22–1.05) | 0.43  (0.21–0.90) | 0.16 |  | 0.23  (0.13–0.63) | 0.40  (0.20–0.92) | 0.65  (0.32–1.28) | <0.001 |
| HOMA-IR, median (IQR) ^d)^ | 1.3  (0.9–2.0) | 1.4  (0.9–2.0) | 1.5  (1.0–2.1) | <0.001 |  | 1.0  (0.7–1.3) | 1.3  (0.9–1.8) | 2.1  (1.5–3.0) | <0.001 |
| BMI, mean (SD), kg/m^2^ | 22.2 (3.1) | 22.9 (3.0) | 23.6 (3.1) | <0.001 |  |  |  |  |  |
| Handgrip strength, mean (SD), kg |  |  |  |  |  | 25.7 (9.7) | 30.9 (10.8) | 33.3 (11.9) | <0.001 |
| Abbreviations: BMI, body mass index；HDL, high-density lipoprotein; METs, metabolic equivalents; hs-CRP, high-sensitivity c-reactive protein; HOMA-IR, Homeostatic Model Assessment for Insulin Resistance.  Handgrip strength levels were classified according to age- and sex-specific tertiles. BMI levels were classified as lean (<18.5 kg/m^2^), normal weight (18.5–24.9 kg/m^2^), and obese (≥25.0 kg/m^2^).  ^a)^ Missing in 1 participant.  ^b)^ Missing in 35 participants.  ^c)^ Missing in 21 participants.  ^d)^ Missing in 65 participants. | | | | | | | | | |

| **Supplemental Table 3. Age- and sex-adjusted incidence (per 1,000 person-years) of cardiovascular disease and its subtypes according to BMI and handgrip strength classification (n = 2,490), 1988–2012.** | | | | | | | | | | | |
| --- | --- | --- | --- | --- | --- | --- | --- | --- | --- | --- | --- |
| BMI levels | Lean  (BMI <18.5 kg/m^2^) | | |  | Normal weight  (BMI 18.5–24.9 kg/m^2^) | | |  | Obese  (BMI ≥25.0 kg/m^2^) | | |
| Handgrip strength levels | High | Medium | Low |  | High | Medium | Low |  | High | Medium | Low  (dynapenic obesity) |
| ***Cardiovascular disease*** | |  |  |  |  |  |  |  |  |  |  |
| No. of events/PYs at risk | 5/569 | 13/754 | 18/1361 |  | 105/11806 | 100/11523 | 108/10022 |  | 45/5605 | 49/3666 | 39/2502 |
| Incidence (95% CI) | 7.9  (0.8-15.0) | 10.9  (4.6-17.2) | 12.8  (6.8-18.7) |  | 10.0  (8.1-12.0) | 9.9  (7.9-12.0) | 13.5  (10.8-16.2) |  | 8.9  (6.1-11.7) | 15.9  (10.9-20.9)* | 20.3  (12.8-27.7)** |
| ***Stroke*** | |  |  |  |  |  |  |  |  |  |  |
| No. of events/PYs at risk | 3/570 | 10/755 | 10/1381 |  | 71/11959 | 65/11677 | 75/10162 |  | 28/5730 | 33/3740 | 29/2533 |
| Incidence (95% CI) | 5.1  (0.0-11.0) | 8.2  (2.8-13.6) | 6.7  (2.5-10.9) |  | 6.7  (5.2-8.3) | 6.2  (4.7-7.8) | 8.9  (6.8-11.0) |  | 5.4  (3.2-7.5) | 10.5  (6.4-14.6) | 16.0  (9.0-22.9)** |
| ***Coronary heart disease*** | |  |  |  |  |  |  |  |  |  |  |
| No. of events/PYs at risk | 2/602 | 3/783 | 10/1424 |  | 46/12237 | 45/11883 | 45/10399 |  | 23/5805 | 21/3864 | 14/2666 |
| Incidence (95% CI) | 2.2  (0.0-5.3) | 2.7  (0.0-5.9) | 6.7  (2.5-10.9) |  | 4.2  (3.0-5.4) | 4.3  (3.0-5.6) | 5.4  (3.8-7.1) |  | 4.4  (2.5-6.2) | 6.8  (3.5-10.0) | 5.3  (2.5-8.2) |
| Abbreviations: BMI, body mass index; PYs, person-years; CI, confidence intervals.  Handgrip strength levels were classified according to age- and sex-specific tertiles. BMI levels were classified as lean (<18.5 kg/m^2^), normal weight (18.5–24.9 kg/m^2^), and obese (≥25.0 kg/m^2^).  *p<0.05, **p<0.01 vs. a reference group (participants with a high handgrip strength and BMI of 18.5–24.9 kg/m^2^). | | | | | | | | | | | |

| **Supplemental Table 4.** **Multivariable-adjusted hazard ratios of development of cardiovascular disease according to handgrip strength levels, 1988–2012** | | | |
| --- | --- | --- | --- |
| Handgrip strength levels | High | Medium | Low |
| **All participants (n = 2,490)** |  |  |  |
| No. of events/at risk | 155/904 | 162/826 | 165/760 |
| HR (95% CI) | 1.00 (reference) | 1.15 (0.92–1.43) | 1.35 (1.08–1.68)* |
|  |  |  |  |
| **Participants aged <65 years (n = 1,794)** |  |  |  |
| No. of events/at risk | 94/681 | 92/602 | 86/511 |
| HR (95% CI) | 1.00 (reference) | 1.08 (0.81–1.45) | 1.30 (0.96–1.75) |
|  |  |  |  |
| **Participants aged ≥65 years (n = 696)** |  |  |  |
| No. of events/at risk | 61/223 | 70/224 | 79/249 |
| HR (95% CI) | 1.00 (reference) | 1.22 (0.86–1.72) | 1.45 (1.03–2.04)* |
| Abbreviations: HR, hazard ratio; CI, confidence interval.  Handgrip strength levels were classified according to age- and sex-specific tertiles. The models were adjusted for age, sex, hypertension, diabetes, serum total cholesterol, serum high-density lipoprotein cholesterol, electrocardiogram abnormalities, current alcohol intake, current smoking habits, regular exercise, and body mass index.  *p<0.05 vs. a reference group (participants with a high handgrip strength). | | | |

| **Supplemental Table 5.** **Multivariable-adjusted hazard ratios of development of cardiovascular disease according to** **BMI levels, 1988–2012** | | | |
| --- | --- | --- | --- |
| BMI levels, kg/m^2^ | Lean  (<18.5) | Normal weight  (18.5–24.9) | Obese  (≥25.0) |
| **All participants (n = 2,490)** |  |  |  |
| No. of events/at risk | 36/163 | 313/1724 | 133/603 |
| HR (95% CI) | 1.04 (0.73–1.49) | 1.00 (reference) | 1.17 (0.94–1.45) |
|  |  |  |  |
| **Participants aged <65 years (n = 1,794)** |  |  |  |
| No. of events/at risk | 12/84 | 172/1243 | 88/467 |
| HR (95% CI) | 1.00 (0.54–1.82) | 1.00 (reference) | 1.27 (0.96–1.67) |
|  |  |  |  |
| **Participants aged ≥65 years (n = 696)** |  |  |  |
| No. of events/at risk | 24/79 | 141/481 | 45/136 |
| HR (95% CI) | 1.06 (0.67–1.66) | 1.00 (reference) | 1.00 (0.70–1.42) |
| Abbreviations: BMI, body mass index; HR, hazard ratio; CI, confidence interval.  The models were adjusted for age, sex, hypertension, diabetes, serum total cholesterol, serum high-density lipoprotein cholesterol, electrocardiogram abnormalities, current alcohol intake, current smoking habits, regular exercise, and handgrip strength. | | | |

| **Supplemental Table 6.** **Subdistribution hazard regression (Fine and Gray) models for development of cardiovascular disease accounting for competing risk of death according to handgrip strength and BMI levels, 1988–2012** | | | | | | | | | | | |
| --- | --- | --- | --- | --- | --- | --- | --- | --- | --- | --- | --- |
| BMI levels | Lean | | |  | Normal weight | | |  | Obese | | |
| Handgrip strength levels | High | Medium | Low |  | High | Medium | Low |  | High | Medium | Low  (dynapenic obesity) |
| **All participants (n = 2,490)** | |  |  |  |  |  |  |  |  |  |  |
| No. of events/at risk | 5/32 | 13/47 | 18/84 |  | 105/595 | 100/588 | 108/541 |  | 45/277 | 49/191 | 39/135 |
| HR (95% CI) | 0.68  (0.27–1.70) | 1.27  (0.70–2.29) | 1.00  (0.60–1.67) |  | 1.00 (reference) | 0.92  (0.70–1.21) | 1.10  (0.84–1.44) |  | 0.84  (0.59–1.20) | 1.30  (0.91–1.86) | 1.50  (1.02–2.21)* |
|  |  |  |  |  |  |  |  |  |  |  |  |
| **Participants aged <65 years (n = 1,794)** | | |  |  |  |  |  |  |  |  |  |
| No. of events/at risk | 2/18 | 4/20 | 6/46 |  | 60/437 | 59/440 | 53/366 |  | 32/226 | 29/142 | 27/99 |
| HR (95% CI) | 0.71  (0.15–3.35) | 1.26  (0.45–3.49) | 0.87  (0.36–2.13) |  | 1.00 (reference) | 0.87  (0.61–1.25) | 1.00  (0.69–1.45) |  | 0.88  (0.57–1.36) | 1.22  (0.77–1.95) | 1.68  (1.05–2.68)* |
|  |  |  |  |  |  |  |  |  |  |  |  |
| **Participants aged ≥65 years (n = 696)** | | |  |  |  |  |  |  |  |  |  |
| No. of events/at risk | 3/14 | 9/27 | 12/38 |  | 45/158 | 41/148 | 55/175 |  | 13/51 | 20/49 | 12/36 |
| HR (95% CI) | 0.67  (0.21–2.18) | 1.34  (0.65–2.77) | 1.13  (0.59–2.15) |  | 1.00 (reference) | 0.93  (0.60–1.43) | 1.22  (0.82–1.81) |  | 0.76  (0.41–1.40) | 1.40  (0.81–2.42) | 1.16  (0.60–2.27) |
| Abbreviations: BMI, body mass index; HR, hazard ratio; CI, confidence interval.  Handgrip strength levels were classified according to age- and sex-specific tertiles. BMI levels were classified as lean (<18.5 kg/m^2^), normal weight (18.5–24.9 kg/m^2^), and obese (≥25.0 kg/m^2^). The models were adjusted for age, sex, hypertension, diabetes, serum total cholesterol, serum high-density lipoprotein cholesterol, electrocardiogram abnormalities, current alcohol intake, current smoking habits, and regular exercise.  *p<0.05 vs. a reference group (participants with a high handgrip strength and BMI of 18.5–24.9 kg/m^2^). | | | | | | | | | | | |

| **Supplemental Table 7. Multivariable-adjusted hazard ratios of development of cardiovascular disease according to handgrip strength levels and the presence or absence of abdominal obesity, 1988–2012** | | | | | | | |
| --- | --- | --- | --- | --- | --- | --- | --- |
| Abdominal obesity | Absent | | |  | Present | | |
| Handgrip strength levels | High | Medium | Low |  | High | Medium | Low |
| **All participants (n = 2,430) ^a)^** |  |  |  |  |  |  |  |
| No. of events/at risk | 83/467 | 97/504 | 99/487 |  | 68/419 | 63/303 | 58/250 |
| HR (95% CI) | 1.00 (reference) | 1.07 (0.80–1.43) | 1.19 (0.89–1.60) |  | 1.01 (0.72–1.42) | 1.30 (0.92–1.85) | 1.56 (1.09–2.24)* |
|  |  |  |  |  |  |  |  |
| **Participants aged <65 years (n = 1,758) ^b)^** | |  |  |  |  |  |  |
| No. of events/at risk | 45/341 | 62/377 | 53/341 |  | 45/326 | 28/213 | 32/160 |
| HR (95% CI) | 1.00 (reference) | 1.13 (0.77–1.66) | 1.18 (0.79–1.76) |  | 1.16 (0.75–1.80) | 1.16 (0.71–1.91) | 1.85 (1.14–2.99)* |
|  |  |  |  |  |  |  |  |
| **Participants aged ≥65 years (n = 672) ^c)^** | |  |  |  |  |  |  |
| No. of events/at risk | 38/126 | 35/127 | 46/146 |  | 23/93 | 35/90 | 26/90 |
| HR (95% CI) | 1.00 (reference) | 0.88 (0.56–1.40) | 1.16 (0.75–1.79) |  | 0.71 (0.40–1.25) | 1.34 (0.80–2.23) | 1.23 (0.71–2.13) |
| Abbreviations: HR, hazard ratio; CI, confidence interval. Abdominal obesity was defined as waist circumference of 90 cm or greater for men and of 80 cm or greater for women. Handgrip strength levels were classified according to age- and sex-specific tertiles. The models were adjusted for age, sex, hypertension, diabetes, serum total cholesterol, serum high-density lipoprotein cholesterol, electrocardiogram abnormalities, current alcohol intake, current smoking habits, and regular exercise. ^a)^ Missing in 60 participants. ^b)^ Missing in 36 participants. ^c)^ Missing in 24 participants. *p<0.05 vs. a reference group (participants with a high handgrip strength and no abdominal obesity). | | | | | | | |

| **Supplemental Table 8. Multivariable-adjusted hazard ratios of development of cardiovascular disease according to handgrip strength levels** **classified by the AWGS cut-off values and BMI levels, 1988–2012** | | | | | | | | |
| --- | --- | --- | --- | --- | --- | --- | --- | --- |
| BMI levels, kg/m^2^ | Lean | |  | Normal weight | |  | Obese | |
| Handgrip strength levels ^a)^ | High | Low |  | High | Low |  | High | Low |
| **All participants (n = 2,490)** |  |  |  |  |  |  |  |  |
| No. of events/at risk | 24/122 | 12/41 |  | 256/1536 | 57/188 |  | 115/559 | 18/44 |
| HR (95% CI) | 1.19 (0.78–1.82) | 1.37 (0.75–2.48) |  | 1.00 (reference) | 1.70 (1.26–2.29)** |  | 1.13 (0.90–1.42) | 1.90 (1.15–3.12)* |
|  |  |  |  |  |  |  |  |  |
| **Participants aged <65 years (n = 1,794)** | |  |  |  |  |  |  |  |
| No. of events/at risk | 10/75 | 2/9 |  | 158/1181 | 14/62 |  | 82/450 | 6/17 |
| HR (95% CI) | 1.13 (0.59–2.17) | 1.21 (0.29–4.98) |  | 1.00 (reference) | 1.70 (0.98–2.96) |  | 1.19 (0.90–1.57) | 3.42 (1.49–7.85)** |
|  |  |  |  |  |  |  |  |  |
| **Participants aged ≥65 years (n = 696)** | |  |  |  |  |  |  |  |
| No. of events/at risk | 14/47 | 10/32 |  | 98/355 | 43/126 |  | 33/109 | 12/27 |
| HR (95% CI) | 1.32 (0.74–2.36) | 1.00 (0.64–2.47) |  | 1.00 (reference) | 1.61 (1.11–2.33)* |  | 0.97 (0.64–1.46) | 1.55 (0.83–2.90) |
| Abbreviations: BMI, body mass index; HR, hazard ratio; CI, confidence interval. ^a)^ Handgrip strength levels were dichotomized using AWGS cut-off values at <28 kg for men and <18 kg for women. BMI levels were classified as lean (<18.5 kg/m^2^), normal weight (18.5–24.9 kg/m^2^), and obese (≥25.0 kg/m^2^). The models were adjusted for age, sex, hypertension, diabetes, serum total cholesterol, serum high-density lipoprotein cholesterol, electrocardiogram abnormalities, current alcohol intake, current smoking habits, and regular exercise.  *p<0.05, **p<0.01 vs. a reference group (participants with a high handgrip strength and BMI of 18.5–24.9 kg/m^2^). | | | | | | | | |
