# Supplementary material for: Association between dynapenic obesity and risk of cardiovascular disease: The Hisayama study
Source: J Cachexia Sarcopenia Muscle. 2024 Oct 8;15(6):2338–48. doi: 10.1002/jcsm.13564 (PMC11634510; doi:10.1002/jcsm.13564)
Supplement: Supplementary file 2 — Figure S1. Flowchart of numbers of included and excluded participants. [file JCSM-15-2338-s002.pptx]

## Slide 1
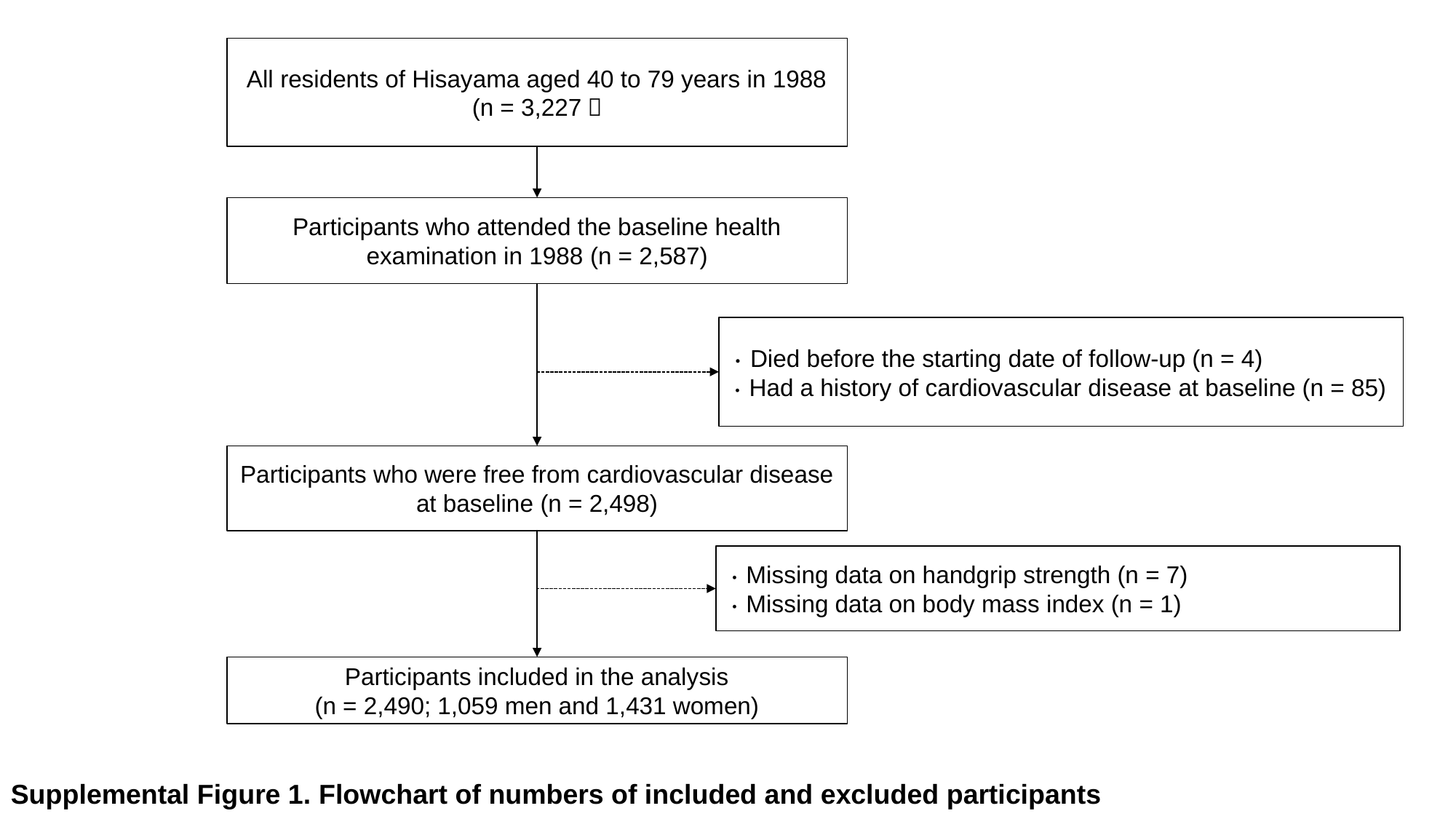

All residents of Hisayama aged 40 to 79 years in 1988
(n = 3,227）
Participants who attended the baseline health examination in 1988 (n = 2,587)
・Died before the starting date of follow-up (n = 4)
・Had a history of cardiovascular disease at baseline (n = 85)
Participants who were free from cardiovascular disease at baseline (n = 2,498)
・Missing data on handgrip strength (n = 7)
・Missing data on body mass index (n = 1)
Participants included in the analysis
(n = 2,490; 1,059 men and 1,431 women)
Supplemental Figure 1. Flowchart of numbers of included and excluded participants
